# Pathways to Progressive Disability in Multiple Sclerosis: The Role of Glial Cells in Chronic CNS Inflammation

**DOI:** 10.1002/glia.70044

**Published:** 2025-05-23

**Authors:** Volker Siffrin

**Affiliations:** ^1^ Experimental and Clinical Research Center, Max‐Delbrück‐Center for Molecular Medicine and Charité–Universitätsmedizin Berlin Berlin Germany; ^2^ Department of Neurology Charité—Universitätsmedizin Berlin, Corporate Member of Freie Universität Berlin and Humboldt‐Universität Zu Berlin Berlin Germany

**Keywords:** astrocytes, chronic inflammation, CNS pathology, glial cells, microglia, multiple sclerosis, neurodegeneration, remyelination

## Abstract

Multiple sclerosis (MS) is the most common non‐infectious inflammatory CNS disease, characterized by progressive neurodegeneration and focal demyelinated lesions. Traditionally considered an autoimmune disease, MS is driven by the immune system's attack on CNS myelin, resulting in cumulative disability. However, conventional anti‐inflammatory treatments often fail to prevent progressive deterioration, particularly in the absence of overt inflammation, highlighting the need for a deeper understanding of its pathogenesis. Recent research has revealed a more complex disease mechanism involving both peripheral immune responses and intrinsic CNS factors, with glial cells playing a central role. Persistent inflammation in MS is associated with mixed active/inactive lesions dominated by microglia and astrocyte dysregulation. These glial populations exhibit maladaptive activation, contributing to failed remyelination and ongoing neurodegeneration. Transcriptomic and epigenomic alterations as well as aging further exacerbate glial dysfunction, creating a self‐perpetuating cycle of inflammation and damage. Emerging evidence suggests that the interplay between peripheral immune cells and glial populations and the potential dual‐use nature of molecular tools shared by the immune system and CNS disrupts homeostatic signaling, leading to a loss of tissue integrity. This review synthesizes findings on glial cell biology in MS, with a focus on microglia and astrocytes, while addressing their roles in demyelination, synapse loss, and neurodegeneration. The limitations of animal models, particularly EAE, in replicating the complexity of MS are also addressed. Finally, critical questions are outlined to guide future research into glial pathology and to identify novel therapeutic approaches targeting progressive MS.

## Main Points

- Multiple sclerosis involves chronic CNS inflammation and neurodegeneration. This review highlights glial cells as key drivers in progressive MS, focusing on lesion pathology, molecular dysfunction, and human‐relevant model systems.

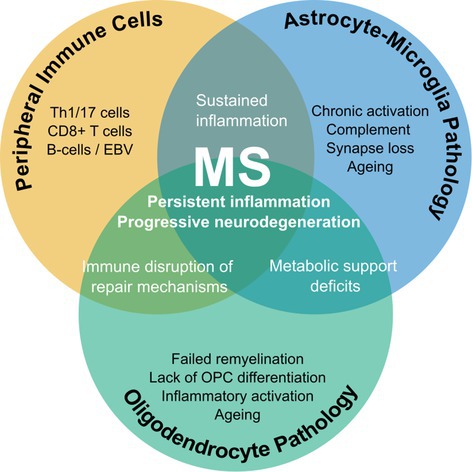

## 1 Introduction

### 1.1 Multiple Sclerosis: An Overview

MS typically begins in young adulthood and affects over 1.2 million people in Europe alone. The disease has a profound impact not only on affected individuals and their families but also on society at large, particularly in terms of socioeconomic burden. CNS pathology in MS is characterized by multiple focal demyelinated lesions, accumulating over time and typically situated around a postcapillary venule and prominently located in specific regions of the CNS, including the periventricular, juxtacortical/cortical, infratentorial areas, the spinal cord, and the optic nerve. MS is widely considered and communicated to be an autoimmune disease that targets CNS myelin, resulting in neurodegeneration and severe disability throughout adult life. According to this autoimmune classification, the immune system is provoked into attacking the normally functioning CNS through a failure to properly discriminate against potential foreign threats and self‐structures. In this paradigm, the CNS is the innocent victim, poorly equipped to cope with attackers wielding the full power of the adaptive immune system. Following this model, treatment strategies for MS seek to continuously suppress peripheral immune cell functions (Comi et al. 2017).

### 1.2 Challenges in MS Research and Treatment

Immunosuppressive therapies are successful in early stages of the disease when inflammatory infiltrates into the CNS are most abundant. However, risks are considerable with opportunistic infections and malignancy as continuous threats in long‐term immunosuppression. Furthermore, MS almost invariably progresses in most patients so that 15–20 years after diagnosis the disease is characterized by accumulating disability and is largely unresponsive to conventional anti‐inflammatory treatments in so‐called progressive MS (Scalfari et al. 2010; Giovannoni et al. 2017). In recent years, the term progressive MS—used to describe patients with worsening disability in the absence of relapse activity usually later in the disease course—has been revisited. A novel concept has been introduced that better reflects the dual pathology of the disease, encompassing both peripheral immune cell‐driven inflammatory disability and a CNS‐secluded inflammatory activity—the latter often without pronounced lymphocyte involvement. These processes are now understood to evolve in parallel rather than sequentially, with significant interindividual variability in the relative contributions of each disease mechanism. Disability resulting from secluded inflammation is now referred to as progression independent of relapse activity (PIRA) (Kappos et al. 2020). Clinically, PIRA can manifest early in the disease and is often refractory to treatment. This recognition of a more complex MS pathophysiology has driven new research aimed at identifying the core mechanisms of non‐self‐limiting, CNS intrinsic inflammation, which would typically subside following clearance of peripheral immune cells. These findings challenge the oversimplified notion of MS as a purely autoimmune disease, carrying significant implications for treatment resistance and the urgent need for novel therapeutic targets. This review explores the potential true nature of MS as a CNS inflammatory disease driven by both immune and CNS cellular factors, resulting in persistent inflammation, neurodegeneration, and progressive disability.

## 2 Who Gets MS? Genetic and Environmental Risk Factors

### 2.1 Genetic Susceptibility

MS is a complex, partially hereditary disease with concordance rates between monozygotic siblings ranging between 25% and 50% (Fagnani et al. 2015). The efforts to determine the mechanisms underlying the cause and progression of multiple sclerosis have been immense, and great insights into certain aspects of neuroinflammation have been achieved. Geneticists spearheaded the movement to determine the underlying genetic disease susceptibility, with some marked successes. Early systematic approaches identified the strong association of MS with the MHC‐II allele HLA‐DRB1*1501 (Stewart et al. 1981), an indication that CD4+ T cells might be involved in disease evolution. The HLA‐DRB1*1501 has been brought into the European population by an eastern pastoralist population about 5000 years ago and is thought to have been positively selected due to protective effects against animal‐borne infections, e.g., *Mycobacterium tuberculosis* (Barrie et al. 2024). By now—in addition to further MHC‐related susceptibility loci—more than 200 non‐MHC susceptibility loci for MS have been identified in large genome‐wide association studies (GWAS) which analyzed genomic data from more than 40.000 MS patients (International Multiple Sclerosis Genetics Consortium (IMSGC) et al. 2013). The theory of MS as a conventional autoimmune disease has been fueled by these results that identified mostly risk variants associated with inflammation‐related genes and accounting for about 50% of MS heritability (International Multiple Sclerosis Genetics Consortium 2019). Some of these genetic MS risk SNPs have been functionally characterized and found to boost pro‐inflammatory T lymphocyte responses (Gregory et al. 2012) but also microglia activation (Consortium 2019; International Multiple Sclerosis Genetics Consortium 2019; Ma et al. 2023). However, for most of the identified candidates, it is unclear at what point in the disease course they exert their influence, which specific processes they influence, and which cell populations are involved in these processes. This indicates that inflammatory mechanisms are not exclusively driven by peripheral immune cells but may also involve tissue‐specific stromal cells, such as glial cells. Beyond disease susceptibility, genetic variants have been recently studied in the context of MS disease severity, revealing a different pattern: most identified SNPs are associated with CNS‐relevant, non‐immune genes (International Multiple Sclerosis Genetics Consortium and MultipleMS Consortium 2023). These factors likely contribute to repair and regeneration following inflammatory damage.

### 2.2 Environmental Factors

In addition to genetic predispositions such as HLA‐DRB1*1501, MS, as a complex genetic disorder, is heavily influenced by environmental factors (Zierfuss et al. 2024). While the strongest evidence exists for Epstein–Barr virus (EBV), additional environmental factors are increasingly recognized as modifiers of disease susceptibility and progression, possibly through epigenetic or inflammatory mechanisms. For instance, geographical latitude during childhood and adolescence has been linked to lower vitamin D levels in regions with reduced sun exposure. Additionally, lifestyle factors including smoking and adolescent obesity significantly contribute to increased MS risk. Dietary factors, potentially interlinked with the microbiome, have also emerged as an important area of investigation due to their possible effects on immune and CNS‐resident cells.

However, the most robustly established environmental risk factor for MS remains seropositivity to EBV. While the Epstein–Barr Virus itself is not the direct cause of MS—it is neither regularly found in the CNS nor exhibits tropism for CNS‐resident cells (Willis et al. 2009; Sargsyan et al. 2010)—its strong association with MS, particularly following infectious mononucleosis (IM), is well established (Thacker et al. 2006). IM, the clinical manifestation of a severe primary EBV infection, has been linked to an increased risk of MS, particularly in individuals carrying the HLA‐DR15 (MHC‐II allele) genetic risk variant (De Jager et al. 2008). A prospective study involving the U.S. Army showed that the risk of developing MS after EBV infection increased 32‐fold (Bjornevik et al. 2022). However, despite the nearly universal seropositivity for EBV (90–95% in the general population), MS remains a rare outcome, highlighting gaps in our understanding of EBV‐induced immune alterations. A complex interplay between EBV, its mechanisms of immune evasion, and genetic variations in both adaptive and innate immunity has been proposed to explain the rare occurrence of CNS autoimmunity following EBV infection (Vietzen et al. 2023). EBV does not universally predispose individuals to autoimmune diseases. However, reproducible associations have been observed between EBV and conditions such as systemic lupus erythematosus (SLE), Sjögren syndrome, and rheumatoid arthritis (RA), suggesting a more nuanced interplay between the virus and immune dysregulation. Like these disorders, MS does not rely on a disease‐specific autoantigen, raising the question of whether an organ‐intrinsic susceptibility factor interacts with EBV‐induced adaptive immune system alterations or if EBV triggers specific changes in organ‐resident cells.

To differentiate between immune system‐derived disease‐promoting factors and target organ‐specific factors, experiences from myeloablative and immune‐ablative therapies in patients offer critical insights. One illustrative example is a trial involving alemtuzumab in patients with highly active progressive MS (Coles et al. 2004). Alemtuzumab is a CD52‐depleting antibody that efficiently depletes B and T lymphocytes. The subsequent lymphocyte repopulation derives from hematopoietic stem cells (Baker et al. 2017). This depletion/repopulation mechanism was marketed as a clean reboot of the lymphoid compartment of the immune system. However, while this treatment was very effective in relapsing MS patients, those with progressive MS kept clinically deteriorating, although without further relapse or MRI activity (Coles et al. 2004). This highlights that the most severe deficits in progressive MS and in patients with prominent PIRA stem from gradual, progressive mechanisms that are not influenced by immune cell depletion. Similarly, MS patients who underwent autologous stem cell transplantation as treatment for progressive MS or allogeneic stem cell transplantation on account of hematological malignancy showed persistent CNS inflammation with demyelination and axonal degeneration (Metz et al. 2007; Lu et al. 2015). These findings underscore a critical point: complete ablation of the peripheral immune system, long considered the driving force behind MS, fails to halt CNS inflammatory disease activity in progressive MS.

## 3 Lessons From Animal Models: Strengths and Limitations of Experimental Autoimmune Encephalomyelitis (EAE)

### 3.1 Insights Gained From EAE

Parallel advancements in (neuro)immunology have elucidated critical aspects of immune cell migration into the CNS and the phenotypic characteristics of autoreactive immune cells that have the capacity to induce neuroinflammation. Much of this understanding stems from studies using EAE, the primary animal model for MS. Consequently, the prevailing perception of CD4+ T cell‐dependent chronic inflammation as the driving force in MS largely originates from EAE research. In EAE, a distinct T cell phenotype–the Th17 cell–plays a critical role in disease manifestation. Key factors include the master transcription factor RORγt which drives Th17 cell differentiation (Ivanov et al. 2006), IL‐23, which stabilizes Th17 cells, and GMCSF, which mediates the manifestation of clinical signs of EAE (McGeachy et al. 2009; Codarri et al. 2011; El‐Behi et al. 2011). Fate mapping studies have revealed that 90% of the pathogenic T cells infiltrating the CNS undergo a phenotypic shift, losing Th17 and gaining Th1‐like functions (Hirota et al. 2011). Th17 cells contribute to a neurodegenerative phenotype through direct neurotoxic effects in EAE (Siffrin et al. 2010). The analysis of transcriptional modifications in CNS Th17 cells in EAE identified key signaling networks and novel molecular targets linked to chronic neuroinflammation, including transcription factors and MS susceptibility genes (Hoppmann et al. 2015).

### 3.2 Limitations of EAE in MS Research

EAE is induced by immunization with myelin antigens and immune stimulants such as pertussis toxin and complete Freund's adjuvant (containing heat‐killed *Mycobacterium* species), which forcibly break natural tolerance. In contrast, neither a unifying “autoantigen” nor a comparable “inflammatory boost” has been identified in MS. The immunization regime induces a strong (Th17) CD4+ T cell response (usually no CD8+ T cell response or B cell/antibody production in the peptide induced models). While myelin antigens are the clear targets in EAE, decades of research have failed to identify common disease‐relevant antigens in MS (Hohlfeld et al. 2016a, 2016b). This stands in stark contrast to autoimmune encephalitides, paraneoplastic brain diseases, and neuromyelitis optica spectrum disorders (NMOSD) and myelin oligodendrocyte glycoprotein (MOG) antibody‐associated disease (MOGAD) (Uzawa et al. 2024), where specific autoantigens such as aquaporin‐4 (AQP4) and MOG have been identified. Both NMOSD and MOGAD are driven by peripherally produced, pathogenic autoantibodies that directly mediate tissue damage. Blocking their production, neutralizing their effects, or eliminating the responsible plasmablasts or their cytokine‐driven activation effectively halts disease activity. This is a fundamental difference from MS, where targeting the peripheral immune system alone is insufficient to prevent progression (Kawachi and Lassmann 2017). In NMOSD, immune responses targeting AQP4 are halted by treating the peripheral immune system, preventing further neurodegeneration. Lesions in NMOSD and particularly MOGAD often show substantial healing, with MOGAD lesions even disappearing over time—an important distinguishing feature from MS (Uzawa et al. 2024). The lack of a unifying antigen in MS is compelling evidence that it differs fundamentally from these primary autoimmune disorders.

Similarly, acute disseminated encephalomyelitis (ADEM)—an uncommon condition primarily affecting children, often in the context of or as a complication of vaccinations—shares more similarities with EAE than with MS. In ADEM, a specific myelin antigen, such as MOG, can often be identified as the target of autoimmune attacks (Höftberger et al. 2020). Unlike MS, ADEM is typically self‐limiting and does not progress to chronic CNS inflammation (Stadelmann and Brück 2004). The absence of CNS‐intrinsic, self‐sustaining inflammation in NMOSD, MOGAD, and ADEM further differentiates these diseases from MS, where independent CNS‐driven immune activity persists even after modifying peripheral immune responses.

### 3.3 The Role of Lymphocytes in MS

In MS, the role of CD4+ T cells and the IL‐23/Th17 axis is less clear‐cut than in peptide‐induced EAE (Friese and Fugger 2009). Acute exacerbations have been linked to increased numbers of Th17 cells (Kebir et al. 2007; Tzartos et al. 2008; Brucklacher‐Waldert et al. 2009), and we have reported that higher numbers of IL‐17 producing T helper cells in the peripheral blood of MS patients treated with natalizumab—a drug inhibiting CNS migration of lymphocytes by blocking α4‐integrin—correlate with the presence of a more destructive lesion phenotype in MRI (Bühler et al. 2016). Conflicting evidence, however, tempers these findings (Hiltensperger and Korn 2018). In contrast to its pronounced efficacy in conditions like psoriasis, targeting IL‐17A alone in MS has demonstrated only modest effects on lesion development, highlighting the complexity of its role in the disease (Havrdová et al. 2016). Although MS is associated with certain MHC‐II variants (and thus CD4+ T cells), oligoclonal CD8+ T cells are more frequently found in active demyelinating lesions in biopsies of MS patients (Babbe et al. 2000; Junker et al. 2007). The role of these CD8+ T cells—whether pro‐inflammatory, suppressive, or incidental—remains unclear (Friese and Fugger 2009). B cells also play a significant role in MS (less so in EAE). B cell depletion therapies effectively reduce inflammatory activity in the relapsing phase of MS and offer some benefit in later stages with ongoing MRI activity (Hauser et al. 2008). However, these treatments fail to stop the progressive deterioration in the absence of overt inflammation (Hawker et al. 2009). Pathogenic B cells in MS are thought to act as antigen‐presenting cells, as no specific autoantibodies have been consistently linked to the disease (Li et al. 2018).

### 3.4 Translational Challenges

The success rate of translating therapies from EAE to MS has been disappointing. While EAE effectively models certain aspects of MS, it does not replicate the disease in its entirety and may differ fundamentally in key areas (Ransohoff 2012). EAE has been invaluable for understanding lymphocyte activation, differentiation, and CNS infiltration, as well as the potential mechanisms of CNS damage mediated by the peripheral immune system (Siffrin et al. 2010; Sorbara et al. 2014; Witte et al. 2019). Additionally, EAE has been instrumental in characterizing CNS endogenous cells, such as astrocytes and microglia, that respond to lymphocyte infiltration and inflammatory mediators. Despite these contributions, EAE cannot reflect the complexity of MS pathogenesis in humans.

Bringing these findings together, it becomes evident that MS is distinct from other autoimmune and inflammatory CNS disorders. The absence of a unifying target antigen, combined with the progressive neurodegeneration observed in MS, sets it apart from classic autoimmune diseases like NMOSD, MOGAD, and ADEM. MS treatment strategies, heavily influenced by EAE research, reflect the autoimmune hypothesis but must be interpreted with caution given the significant differences between the human disease and its animal model.

### 3.5 Human‐Centered Research and Alternative Approaches in Experimental Models

Advancements in multi‐omics technologies and high‐resolution single‐cell analysis have significantly enhanced our ability to extract meaningful insights from rare and precious primary tissue samples (Kooistra and Schirmer 2025), offering an unprecedented level of resolution into cellular and intercellular dynamics (Schirmer et al. 2019; Absinta et al. 2021; Chomyk et al. 2024; Macnair et al. 2025) and epigenetic information (Lee et al. 2024). These innovative approaches collectively provide new avenues to investigate the complexities of MS pathophysiology beyond the limitations of traditional models.

With the advent of induced pluripotent stem cells (iPSCs), the toolbox for neuroscientific research has been expanded, offering powerful alternatives or additions to traditional animal models. Human‐derived disease‐relevant biosamples and cells provide a unique advantage, enabling the study of human glial cell biology (Hasselmann and Blurton‐Jones 2020), extreme clinical disease phenotypes (Alisch et al. 2021; Kerkering et al. 2023) and the effects of genetic risk factors on CNS‐intrinsic cells (Lee et al. 2025; Ovesen et al. 2025). This is particularly relevant as emerging genetic engineering tools, such as CRISPR‐Cas9‐based transcriptional modification and modulation, have further expanded the capabilities of iPSC research models. A nuclease‐deficient “dead” (d)Cas9 fused to either a transcription activator (e.g., Viral Protein R, VPR; CRISPRa) or a transcription repressor (e.g., Krüppel‐associated box, KRAB; CRISPRi) enables precise gene regulation (Schoger et al. 2020, 2021).

Additionally, the development of increasingly sophisticated 3D CNS organoid models offers a promising avenue for addressing unresolved questions in neuroscience. While these organoid‐based approaches have so far been most impactful in studying neurodevelopmental (Jourdon et al. 2023) and monogenetic disorders (Lisowski et al. 2024; Matusova et al. 2025), they are now emerging as valuable tools for investigating diseases of the mature nervous system. Overcoming current challenges, such as achieving multi‐lineage differentiation (Faustino Martins et al. 2020; Sabate‐Soler et al. 2022) and vascularization (Kistemaker et al. 2025), will be crucial to fully exploit their potential in modeling complex neurodegenerative and inflammatory processes.

After all, the physiology of a living organism seems essential for validating the relevance of in vitro findings. To bridge this gap, humanized in vivo models have been proposed. These models generally fall into two categories: (i) genetic humanization, where single or multiple human genes are introduced into the mouse genome, and (ii) cellular humanization, where human cells are xenotransplanted into immunocompromised mice to enable functional integration and study human‐specific biological processes. While immune‐humanized mice, particularly for major MS risk‐associated genetic variants (primarily genes linked to immune function), have existed since the late 1990s (Madsen et al. 1999), models incorporating CNS cell‐ and function‐relevant humanization were lacking until recently. Xenotransplanted mice, for example, those engrafted with human microglia (Mancuso et al. 2024) or astrocytes (Goldman et al. 2015), have been used to study human‐specific aspects of disease. Key studies now also demonstrate that the transplantation of human organoids into a mouse brain can lead to proper vascularization and integration within the host organism (Kelley et al. 2024).

By integrating high‐resolution insights from primary disease‐relevant tissues with the advancements in human‐derived and humanized models using computational neuroscience and artificial intelligence (AI)‐based tools, researchers can gain a more comprehensive understanding of the intricate cellular interactions underlying complex disorders. This convergence of cutting‐edge technologies not only bridges the gap between in vitro and in vivo studies but also enhances the translational potential of experimental models, ultimately paving the way for more precise and human‐relevant therapeutic strategies.

## 4 Revisiting the Pathogenesis of MS

**FIGURE 1:**
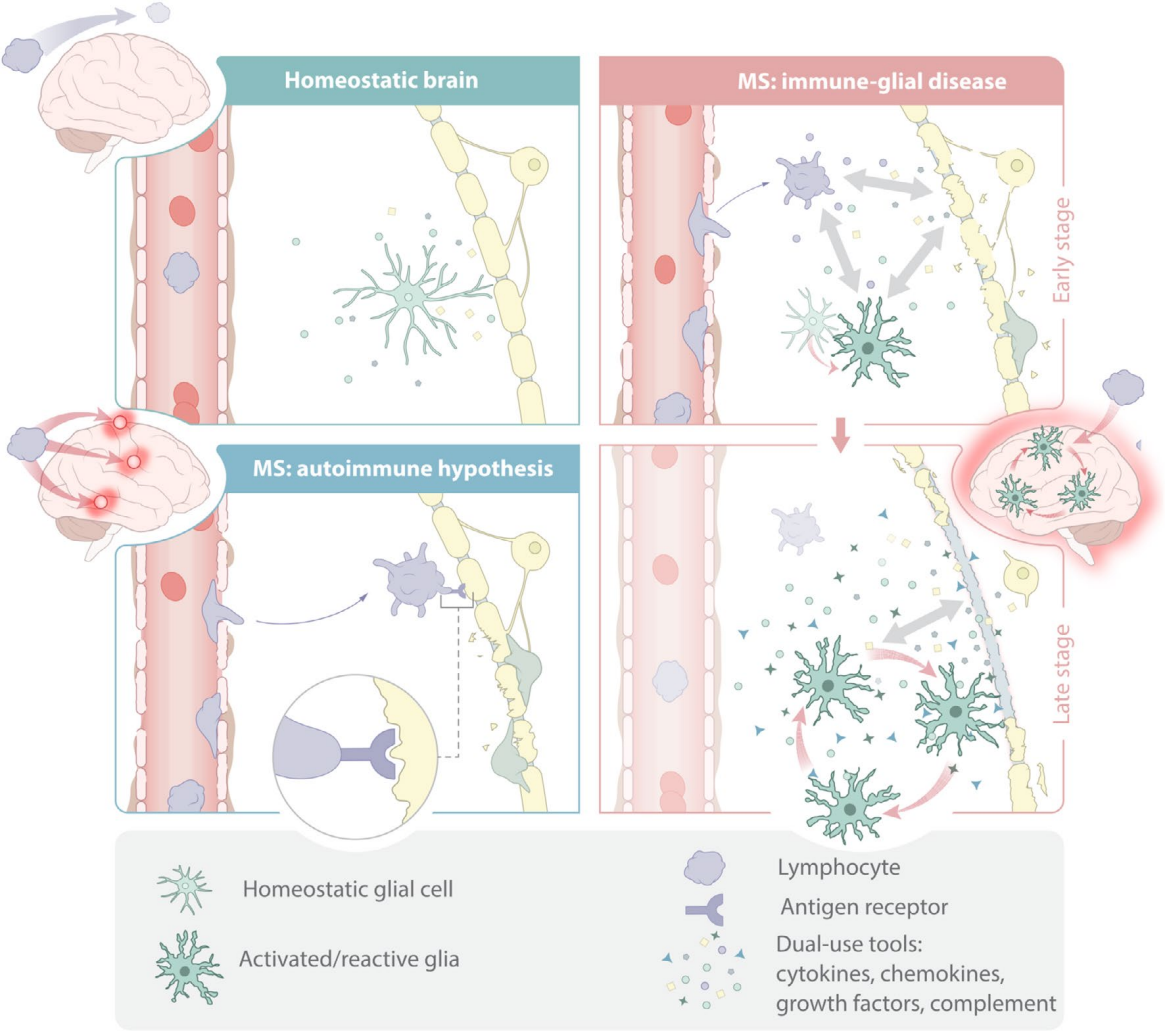
Concept of MS disease pathology: Homeostatic Brain: In the homeostatic brain, glial cells maintain neuronal viability in part through use of mediators that are also found in the immune system. The peripheral immune system is not entering the parenchyma. MS: Autoimmune hypothesis: Antigen directed attack carried out by CNS reactive lymphocytes causes demyelination and destruction of the BBB. MS: Immune‐glial disease: Initial neuroinflammation (possibly mediated in part by peripheral immune cells) alters expression programmes of CNS cells. Altered CNS cells give rise to survival niches for peripheral immune cells and effect continuous demyelination and inflammation through misapprehension of dual‐use immune‐neural mediator signaling.

The finding of progressing neurodegeneration despite severe immunosuppression has been the source of great controversy. The prevailing explanation for failure of immunosuppressive treatment is the potential existence of a currently unknown “non‐inflammatory” component of the disease (Herndon 2002). Some researchers propose that MS might primarily be a neurodegenerative disease—potentially originating in oligodendrocytes or neurons—which then triggers secondary peripheral immune responses (Stys 2013). In this view, inflammation is considered an epiphenomenon possibly accelerating the neurodegenerative course but not driving the disease itself. However, this hypothesis requires a clear genetic, non‐immune system basis, which could not be found in the large genome‐wide association studies (International Multiple Sclerosis Genetics Consortium (IMSGC) et al. 2013; International Multiple Sclerosis Genetics Consortium 2019). Most proponents of the traditional autoimmune paradigm describe the progressive stage of MS—characterized by the absence of relapses and minimal signs of inflammation on MRI—as reflecting a “neurodegenerative feature” of the disease, potentially explained by “insufficient neuronal reserve capacity” (Giovannoni et al. 2017). However, this explanation does not align with histopathological evidence of ongoing inflammation in these patients (Prineas et al. 2001; Kutzelnigg et al. 2005; Stadelmann et al. 2011). A third perspective suggests a more intricate model, wherein the initial autoimmune response—possibly driven by an EBV‐triggered, overactive immune response targeting CNS antigens—progresses into a chronic condition. This chronic phase could arise from the target organ's intrinsic susceptibility to fail in containing innate or intrinsic inflammatory processes, which no longer depend on peripheral triggering factors (Figure 1).

## 5 Glial Pathology in MS

### 5.1 Lesion Morphology and Development

At this point, it is relevant to recapitulate the insights gained on MS lesion morphology and development over the last 150 years since the first detailed descriptions in the 19th century. On a microscopic level, neuropathologists differentiate between active MS lesions, mixed active/inactive lesions, and chronic inactive, hypocellular white matter lesions, which are often found in parallel in the same patient. Furthermore, there are distinct cortical and gray matter lesions. Active lesions are defined by a lesion‐centric vein which is surrounded by peripheral immune cells (“perivascular cuffing”) from there, demyelination is extending centripetally with a looser interception of lymphocytes and myelin‐laden phagocytes. Characteristic of MS—and most prevalent in progressive MS—is the mixed active/inactive lesion (Luchetti et al. 2018), which is marked by predominant inflammatory activity at the lesion rim, i.e., parenchymal border of the lesion toward the normal‐appearing white matter (centrifugal lesion activity). Mixed active/inactive lesions show some phagocytic activity with up‐regulation of inflammatory markers such as MHC‐II, morphological changes in microglia, and (rare) findings of myelin deposits in the cytoplasm. Active lesions get rarer as the disease progresses, while mixed active/inactive lesions are frequent even decades since onset of chronic disease progression (Prineas et al. 2001; Kutzelnigg et al. 2005).

### 5.2 Blood–Brain Barrier Dysfunction: Temporary Disturbance or Persistent Feature of MS Pathology?

The blood–brain barrier (BBB) and the perivascular space constitute two anatomically distinct interfaces between the endovascular compartment and the CNS parenchyma (Bechmann et al. 2007). This distinction is critical, as (early) active MS lesions typically present with BBB disruption, characterized by the passive leakage of soluble factors–such as plasma proteins–into the brain parenchyma. This leakage predominantly occurs at the capillary level and is clinically exemplified by MRI contrast enhancement, frequently observed within the first weeks following the emergence of a new lesion (Miller et al. 1993) and strongly correlates with large‐scale immune cell infiltration (Brück et al. 1997).

In contrast to BBB leakage, immune cell migration into the perivascular spaces is an active, tightly regulated process involving multiple steps: immune cell arrest, activation, and subsequent transmigration (Mapunda et al. 2022). Notably, this cellular migration can occur even in the absence of overt BBB leakage. While BBB leakage is primarily an early and transient event in MS, much of the disease's pathophysiology unfolds behind a macroscopically intact BBB. Nevertheless, accumulating evidence suggests that BBB‐associated cell types—including astrocytes, endothelial cells, pericytes, and perivascular macrophages—undergo persistent molecular and functional changes, particularly during progressive stages of the disease (Claudio et al. 1995). The pro‐inflammatory potential of endothelial cells, including their capacity to produce cytokines and chemokines, is well established (Prat et al. 2001).

The perivascular space functions as a critical interface where infiltrating immune cells interact with resident cells as pericytes and perivascular macrophages. These resident cells interpret environmental cues and orchestrate appropriate responses, for example during brain infections (Owens et al. 2008). This compartment is markedly altered in active MS lesions, and structural abnormalities persist even in remyelinating lesions (Brosnan and Raine 2013). Notably, incomplete coverage of the parenchymal basal lamina by astrocytic end feet has been documented, suggesting ongoing dysfunction of the glia limitans and a failure to fully restore homeostasis.

Moreover, certain MS pathologies–such as cortical lesions associated with meningeal inflammation–may involve entirely distinct mechanisms of immune cell infiltration. This is partly due to the different vascular architectures of the meningeal vessels and subarachnoid space compared to the brain parenchyma (Owens et al. 2008).

### 5.3 Mechanisms of Demyelination

A unifying feature across lesion types is the near‐complete demyelination of the lesion area. However, the precise mechanism of demyelination remains debated. Reports describe vesicular dissolution as evidence for a humoral etiology (Périer and Grégoire 1965; Raine et al. 1999; Weil et al. 2016), while others highlight myelin stripping by phagocytes as a cellular mechanism, particularly in early and highly inflammatory lesions (Yamasaki et al. 2014).

Oligodendrocytes are commonly depleted in MS lesions and show MS‐specific alterations also in normal appearing white matter (Jäkel et al. 2019); oligodendrocyte precursor cells (OPCs) are often present near and in the lesions; OPC proliferation and migration are common events (Raine et al. 1981; Wu and Raine 1992). Remyelination activity is frequently found alongside active demyelination (Prineas et al. 1993; Raine and Wu 1993) and seems to persist for decades. However, this remyelination rarely succeeds in fully restoring properly myelinated axons within previously demyelinated areas.

### 5.4 Failed Remyelination

Remyelination activity tends to decrease with both patient age and lesion age, is predominantly observed in active lesions, and may even fail in the context of preserved oligodendrocytes (Klotz et al. 2023). Since axons depend heavily on myelination for their trophic support, prolonged demyelination can lead to axonal loss and subsequent accumulation of disability over time (Dubois‐Dalcq et al. 2008). Lesion location also plays a significant role in remyelination efficiency, with cortical lesions exhibiting more active remyelination (Albert et al. 2007), which is associated with reduced neuroaxonal damage (Lazzarotto et al. 2024). For an in‐depth analysis of demyelination in MS and other neuroinflammatory disorders, readers are directed to comprehensive reviews on that matter (Lassmann 2014, 2020). Similarly, foundational details on oligodendrocytes and myelin can be found in works by Simons et al. (Simons et al. 2024). Remyelination varies significantly both within and between MS patients, without any clear pattern (Patrikios et al. 2006; Goldschmidt et al. 2009). Some lesions undergo perpetual cycles of remyelination and re‐demyelination, though the underlying drivers remain unclear. This observation suggests that the challenge in MS is not primarily one of OPC or oligodendrocyte survival but rather the creation of appropriate conditions for remyelination. This is specifically challenging in lesions with persistent inflammatory activity, making remyelination seem like a Sisyphean task. It is crucial to note that oligodendrocytes not only insulate white matter axons but also provide metabolic support. This dual function has been demonstrated in mice with defective myelin basic protein, a key protein required for compacted myelin sheets (Griffiths et al. 1998). In inactive lesions and the cores of mixed active/inactive lesions, the absence of mature oligodendrocytes (Heß et al. 2020) likely contributes to progressive neuroaxonal loss. One of MS's most distinctive features is the widespread failure of successful remyelination, even in patients who are effectively treated for relapse prevention and do not develop new MRI lesions. This failure occurs across the majority of lesions and contrasts sharply with both animal models of MS (Lassmann 2020) and other demyelinating disorders, such as MOGAD, where nearly complete remyelination of previously demyelinated areas is typical (Höftberger et al. 2020; Sechi et al. 2021). The persistence of lesions with preserved axons but without remyelination, despite the nearby presence of largely intact OPCs and oligodendrocytes, suggests highly localized mechanisms that inhibit regeneration. Transcriptomic studies of MS lesions have identified distinct changes in microglia and astrocytes, termed MIMS (microglia inflamed in MS), “immune‐like” OPC, “stressed” oligodendrocytes, and AIMS (astrocytes inflamed in MS), with complement factor C1q and other members of the complement system emerging as a key mediators (Absinta et al. 2021).

### 5.5 Evolutionary Insights: Myelination and Human Brain Development

In animal models, developmental myelination has been shown to restrict later neuroplasticity while protecting neuroaxonal structures (Xin et al. 2024). In humans, brain development has led to specific adaptations in CNS myelination. While developmental myelination begins around birth, like in other species, it extends late into adolescence and even beyond. This prolonged process is a hallmark of human evolution. Oligodendrocytes and their myelin sheaths have evolved functions that go well beyond the electromechanical insulation of axons (Stadelmann et al. 2019). Adaptive myelination allows for fine tuning of electrical signal transmission within hardwired neuronal networks by forming myelin patches—often with long internodes or gaps of unmyelinated axons. In humans, this process predominantly affects cortical areas which have been expanded with human evolution (e.g., the pre‐frontal cortex) and continues well into the 30s and 40s, differentiating humans from other primates, where it typically concludes by the end of adolescence (Wang and Young 2014). For these dynamic processes, feedback loops and precise molecular cues are essential to regulate rapid myelination and demyelination. Exploring the mechanisms of the human‐specific extended period of myelination and demyelination, which is evolutionarily recent, may offer insights into the underlying mechanisms of MS. Understanding the roles of other glial cells, such as microglia and astrocytes, is equally important. Research indicates that while microglia are dispensable for developmental myelination, they are essential for myelin maintenance, i.e., myelin integrity and adequate sizing. For instance, individuals with a heterozygous mutation in CSF1R, which leads to reduced microglial numbers, exhibit hypermyelinated and dysmyelinated axons, highlighting microglia's critical role in preserving adequate myelin sheaths (McNamara et al. 2023). However, the molecular processes remain poorly understood.

### 5.6 Astrocytes: From Homeostatic Support to Neuroinflammatory Reactivity

Astrocytes are central to synapse formation and pruning, working in cooperation with microglial cells to promote neuronal network formation (Christopherson et al. 2005; Chung et al. 2013). They are also critical components of the modern synapse, forming the so‐called tripartite synapse that facilitates neurotransmitter shuttling and electrical activity regulation (Perea and Araque 2007). Astrocytes are implicated early in MS pathogenesis—perhaps even before oligodendroglial damage—and play diverse roles in damage and repair in MS lesions (Brosnan and Raine 2013). In the white matter, astrocytes contribute to the energy supply of the axono‐oligodendroglial unit interacting with oligodendrocytes via gap junctions to maintain myelin sheath integrity (Tress et al. 2012). Human astrocytes differ from their rodent counterparts, being larger and more morphologically complex, with an enhanced capacity to support neuronal networks. Transplantation of human astrocytes into mice improved cognitive performance, suggesting evolutionary adaptations for the human brain's neuronal density (Han et al. 2013). Under inflammatory conditions, astrocytes may also directly support axonal energy demand, although the extent of this support remains unclear. In MS biopsies, demyelinated axons have been described as forming unusual membranous communications with astrocytes, suggesting a metabolic role beyond their recognized function in scar formation (Raine 1978). Depleting astrocytes in EAE resulted in increased immune cell infiltration and exacerbated clinical signs, underscoring their importance in modulating inflammation (Voskuhl et al. 2009). We found critical involvement of astrocytes in neuroprotective programmes in EAE and MS (Alisch et al. 2021; Kerkering et al. 2023; Rosiewicz et al. 2023). Conversely, astrocytes can also drive inflammation and neuronal damage through autocrine glycolipid‐mediated activation (Mayo et al. 2014).

With the specialization of the brain for high plasticity in function, cellular interdependence has emerged, particularly between oligodendrocytes and other CNS cells, such as astrocytes (Williams et al. 2007). Astrocytes play a critical role in supporting oligodendrocytes, providing essential metabolic support. The strong dependence of oligodendrocytes on astrocytes is highlighted in conditions like Neuromyelitis optica spectrum disorders (NMOSD), an autoimmune astrocytopathy where astrocyte depletion, often mediated by autoantibodies targeting aquaporin‐4 (AQP4), leads to complete demyelination in affected lesions (Hinson et al. 2012).

### 5.7 Microglial Dynamics: From Network Shapers to Inflammatory Phagocytes

Microglia arise from embryonic yolk sac‐derived cells and maintain a self‐sustaining population independent of bone marrow‐derived cells (Ginhoux et al. 2013). In homeostasis, they prune synapses, clear debris, and support neuronal network modification, processes critical for learning and memory (Schafer et al. 2012; Parkhurst et al. 2013). Disruption of these roles is linked to neurological diseases (Sellgren et al. 2019). When activated by injury or pathology, microglia transform rapidly, retracting processes and adopting an amoeboid morphology capable of migration, proliferation, and release of substances that influence the pathological process (Hanisch and Kettenmann 2007). These perturbations can lead to state changes of these cells from a surveying state to various states of cellular stress (Sinner et al. 2023). Emerging evidence from deep sequencing reveals that microglial activation is not binary but highly stimulus‐dependent, multifaceted, and distinct from peripheral macrophage responses (Masuda et al. 2019; Sankowski et al. 2019).

These findings suggest that microglia, like astrocytes, have the potential to transition into a chronic inflammatory state that might underly the pathology observed in MS lesions. Astrocytic alterations have been noted in MS since the earliest histopathological descriptions. However, the characterization of “reactive” astrocytes and “activated” microglia in MS remains superficial, primarily describing cytoskeletal changes (Sofroniew and Vinters 2010). In this context, recent progress in MS treatment has begun to reflect a shift toward targeting CNS‐resident immune mechanisms. Notably, the first successful phase 3 clinical trial of the Bruton's tyrosine kinase inhibitor (BTKi) tolebrutinib has demonstrated a clear effect on disability progression in a trial in non‐relapsing secondary progressive MS (Fox et al. 2025). This effect is most likely mediated through the direct modulation of activated microglia, rather than through peripheral immune suppression (Gruber et al. 2024). Tolebrutinib is the first therapeutic agent shown to slow disease progression in people with MS in the absence of overt inflammatory activity, marking a significant milestone in the treatment of progressive MS.

### 5.8 Oligodendrocyte Lineage Cells: Beyond Myelination

Accumulating evidence supports the idea of major alterations in glial subsets associated with chronic MS lesions. Notably, inflammatory gene upregulation is observed not only in microglia but also in astrocytes and oligodendrocyte lineage cells (Table 1), particularly oligodendrocyte precursor cells (OPCs). OPCs exhibited a distinct gene expression profile, strongly expressed multiple growth factor receptors, including FGFR1, EGFR, and PDGFRB, indicating responsiveness to mitogenic and differentiation signals. They also upregulated genes such as VEGFA, BDNF, and IGF2BP2, which are involved in vascular interaction, neuroprotection, and cell survival. Importantly, OPCs expressed MHC class I molecules (HLA‐A, HLA‐C), as well as immune‐related receptors like IL1RAP and IL17RB, suggesting a potential for immunological crosstalk during neuroinflammation. Expression of TNFRSF21 further supports the idea that OPCs can respond to pro‐inflammatory cues, which may influence their differentiation or contribute to vulnerability in diseases like MS. Overall, this expression pattern highlights the multifaceted role of OPCs in MS as both progenitors for myelinating cells and active participants in the persistence of the inflammatory process.

**TABLE 1:** Selected differentially expressed genes identified in transcriptomic studies of MS lesions, compiled from previously published datasets (Schirmer et al. 2019; Absinta et al. 2021; Macnair et al. 2025).

	Macnair et al. 2025	Absinta et al. 2021	Schirmer et al. 2019
HLA‐type I			
HLA‐A	OPC_1 (++), OPC_2 (+), COP (+), Oligo_F (+) Astro_A (AGC, +), Astro_Mixed (++) Astro_Oligo (+) Micro_C/E (MIMS, +), Micro_Prolif (+), Micro_Astro (HM, +) EC Capillary (+), EC Venous (+), Pericyte (+), Endo_PVM (+) PT (+)	EC (+), OPC (+)	EC (+++), PT (++), BP (+++), EN‐L5‐6 (+)
HLA‐B	Astro_Mixed (+++) Micro_A (HM, +), Micro_C/E (MIMS, +), Micro_Prolif (+), EC_Capillary (+), EC_Venous (+), Pericyte (+), Endo_PVM (+) PT (+)	EC (+++), PI (+++)	
HLA‐C	Astro_Mixed (++), Astro_Oligo (++) Micro_C/E (MIMS, +), Micro_Prolif (, +), Micro_Astro (HM, +) EC_Capillary (+), EC_Venous (+) Pericyte (+) PT (+)	EC (++), OPC (+), chronic active lesion edge (+)	Endo cells (+++), PT (++), BP (+++), EN‐L5‐6 (+)
HLA‐E	Astro_C (+), Astro_D (RA, ++), Astro_F (RA, +), Astro_Mixed (+++) Astro_Oligo (, +), Micro_A (HM, +) Micro_C/D/E (MIMS, +), Micro_Prolif (+), Micro_Astro (HM, +), EC_Capillary (++), EC_Venous (, +), EC_Arterial (+), Pericyte (+)	EC (++), PI: (+++) AIMS (++) Chronic active lesion edge (+)	EC (+++), BP (++)
HLA‐type II			
HLA‐DPA1	Micro_A (HM, +), Micro_C/E (MIMS, +), Endo_PVM (+++)	OL (++), control white matter (+)	
HLA‐DPB1	Micro_A (HM, +), Micro_C (MIMS, +), Micro_E (MIMS, +), PVM (+) Endo_PVM (+++)	Control white matter (+)	
HLA‐DQB1	Micro_C/E (MIMS, +)		
HLA‐DRA	Astro_Mixed (+++), Micro_A (HM, +), Micro_C/E (MIMS, +), Micro_Astro (HM, +), Endo_PVM (+++)	AIMS (+++), MIMS (++), OL (++)	Microglia (++)
HLA‐DRB1	Astro_Mixed (+++), Micro_A (HM, +), Micro_C/E (MIMS, +), PVM (+), EC_Venous (+) Endo_PVM (++)	AIMS (+)	Microglia (++)
Chemokines/Chemokine receptors			
CXCL10	Micro_A (HM, +++), Micro_C (MIMS, +), Micro_D (MIMS, +++)		
CX3CL1	Astro_A/B/C/D/F (+), Astro_E (+++)		
CCL5	Micro C (MIMS, +), Micro E (MIMS+++)	VC++, PT (+++)	
Growth factors/receptors			
BDNF	Exc_THEMIS_B (+++)		
CNTFR	Astro_A (AGC, +), Astro_B (AGC, +)		
EGFL7	Exc_CUX2_RORB_C (+), EC_Capillary (++), EC_Venous (+), EC_Stressed (+), Pericyte (+)	PI (+++), HM (+)	EC (+++)
TGFBR1	COP (OL, +++), Astro_Mixed (OL, ++), Micro_A (HM, +), Micro_C/D/E (MIMS, +), Micro_Prolif (+), Endo_PVM (+), PT (++)	AIMS (+++), MIMS (++), AGC (++), vascular cells (+), lesion core+	Phagocytes (++), Microglia (+++)
TGFBR2	Astro_Mixed (+++), Micro_A/B (HM, +), Micro_C/D (MIMS, +) EC_Capillary (+), EC_Venous (+), EC_Stressed (+), Perivascular_Fibroblast (+)	AIMS (++), PI (++), M (++), VC (++), OL (++), vascular cells (+), periplaque+	Microglia (++), Endo cells (++), Stromal cells (++)
TGFBR3	Astro_A/B (AGC, +), Astro_D (RA, +) Inh_SST_B (+), Inh_VIP_A (+), Inh_LAMP5_B (+) EC_Venous (+), Perivascular_Fibroblast (+++), PT (+)	PI (+), M (+++), VC (+)	
TGFB1		vascular cells (+), control white matter (+)	
TGFB2	Astro_A (AGC, +), Astro_D/F (RA, +), Inh_VIP_A (++), Inh_LAMP5_A (+), Inh_RELN_A (+), EC_Venous (++)	MDC (++)	Astrocytes (++)
VEGFA	OPC_1 (+++), Astro_A (AGC, +), Astro_D/F (RA, +), Astro_Mixed (+)	DOL (++), HM (+), periplaque (+)	Astrocytes (++)
VEGFC	EC_Capillary (+), EC_Arterial (OL, +++), Arterial_Smooth_Muscle_Cell (++)	PT (++)	
FGF14	OPC_1/2 (+++), COP (+) Astro_D (RA, +), Astro_E (RA, ++), Astro_Ciliated (RA, +++) Exc_CUX2 (+), Exc_CUX2_RORB_B (+), Exc_RORB_A (+), Exc_TLE4_A/B/C (+), Inh_VIP_B (+), Inh_LAMP5_A/B (+), Inh_RELN_A/B (+), Inh_RELN_B (+)	PT (+++), OL (+++), BP (++), FLC (++), AIMS (+++), PVM (+++)	IN‐SST (++), OPC (+++), EN‐MIX (+++), IN‐VIP (++), IN‐SV2C (+++), OL (++)
FGF12	OPC_1 (+++), OPC_2 (+++), COP (+++), Exc_CUX2 (+), Exc_CUX2_RORB_B/C (+), Exc_RORB_A/B (+), Exc_TLE4_A (+), Inh_Pvalb_A (++), Inh_Pvalb_B (+), Inh_Pvalb_C (+), Inh_SST_A (+), Inh_RELN_A (+)	PT (+++), OL (+++), FLC (++), AIMS (+++)	IN‐PVALB (+++), IN‐SST (++), OPC (+++), EN‐MIX (++)
FGFR1	OPC_1/2 (+++), Oligo_G (DOL, +) Oligo_Astro (OPC, +) Astro_D/E/F (RA, +), Astro_E (RA, +), Astro_Ciliated (RA, +), Astro_Mixed (OL, +), Exc_RORB_A (+), Exc_TLE4_A/C (+++), Inh_Pvalb_A (+) EC_Capillary (+), EC_Arterial (+) EC_Stressed (++), Pericyte (+) Arterial_Smooth_Muscle_Cell (+) Perivascular_Fibroblast (+)	DOL (+), OPC (+), HM (+), periplaque+	
MEGF11	OPC_1 (+++), OPC_2 (+++), COP (+++), Exc_TLE4_B (+++), Inh_VIP_B/D (+++), Inh_RELN_A	OL (+++), AIMS (+++)	OPC (+++)
EGF	Astro_D (RA, +++)		
EGFR	OPC_1/2 (+++), COP (+) Astro_A/B (+), Astro_E (RA, +) Inh_VIP_A/D (+), Inh_VIP_B (++), Inh_LAMP5_A (++), Inh_LAMP5_B (+), Inh_RELN_A (+++), EC_Capillary (+), EC_Venous (+), EC_Stressed (+), Pericyte (+)	OL (++), RA (+), MOL (++), MDC (+++)	IN‐VIP (++), IN‐SV2C (+++)
PDGFRB	Astro_A (AGC, ++), Astro_B (AGC, +), Pericyte (+++), Arterial_Smooth_Muscle_Cell (+)	MOL (+++), OPC (+++), AIMS (+)	Stromal cells (++)
IGFBP7	Astro_D/F (RA, ++), Astro_mixed (+), Endocyte_Capillary (+), Endocyte_Stressed (+), Pericyte (++), Arterial_Smooth_Muscle_Cell (+)	RA (++), PI (+++), MOL (+++), MDC (++), DOL (++), AIMS (+)	Astrocytes (++), Endo cells (+++), Stromal cells (+++)
IGFBP5		RA (+), M (++), AIMS (+)	
IGFBP4	Endocyte_Arterial (+), Pericyte (+), Arterial_Smooth_Muscle_Cell (+++), Perivascular_Fibroblast (+)	MOL (+)	
IGFBP3	EC_Capillary (+), EC_Venous (+), EC_Arterial (+++)	PI (++), PT (+)	
**IGFBP2**	Perivascular_Fibroblast (+++)	MOL (++)	
**IGF2BP2**	COP (+++), EC_Venous (++)	HM (++)	
Cytokines/cytokine receptors			
IFNGAS1		MIMS (+++), VC (+++)	
IL1b	Astro D (RA, +++), Astro F (RA, +), Micro A (HM, +)		
IL1R1	Astro_F (RA, +++) Endocyte_Venous (+++), Pericyte (+), Perivascular_Fibroblast (+)	PI (++), OL (++), HM (+), perivasMac (+)	
IL12RB2	Exc_RORB_A (+), Exc_THEMIS_RORB (++), Exc_THEMIS_A (+), Inh_VIP_A (++), Inh_VIP_B (+++), Inh_VIP_D (+++)	VC++	
IL17RA	Micro_A/B (HM, +), Micro_E (MIMS, +), EC_Capillary (+), Endo_PVM (+) PT (+)		
IL17RB	OPC_1 (, +++), Astro_A (AGC, +), Astro_C/E (RA, +)		Astrocytes (+)
IL15	Micro_C (MIMS, ++), PVM (++)	AIMS (+), lesion core	
IL18	Micro_A/B (HM, +) Micro_B (HM, +), Micro_E (MIMS, +), Micro_Prolif (+)	AIMS (++), MIMS (+), HM (+)	Microglia (++)
IL18R1	Endocyte_Capillary (+), Endocyte_Venous (+), Endocyte_Stressed (+), PT (+)	PI++, VC+, homeostatic microglia+	
IL10RA	Micro_C (MIMS, +), PVM (+), PT (+++)		
IL1RAP	OPC_1/2 (+++), Astro_Mixed (++) Micro_A (HM, +), Micro_C (MIMS, +), Micro_Prolif (+) Exc_CUX2_RORB_B (++), Exc_RORB_B (+++), Inh_Pvalb_A (++), Inh_Pvalb_B (+), Inh_VIP_A (+), Inh_VIP_B (+), Inh_LAMP5_A (+++)	AIMS++, MIMS+, chronic active edge (+)	
IL1RAPL1	Oligo_A/B/C/E/F/G/E (+), Astro_Oligo (+++) Micro_Oligo (+++) Exc_CUX2_RORB_A (+), Exc_CUX2 (+), Exc_CUX2_RORB_B (+), Exc_CUX2_RORB_C (+) Exc_THEMIS_B (+), Exc_TLE4_A (+) Exc_TLE4_C (+), Neuro_Oligo (+), Inh_Pvalb_A (+) Inh_VIP_B (+), Inh_LAMP5_A (++), Inh_LAMP5_B (+) Endo_Mixed (+++), Endo_Oligo (+++)	Perivascmac (+++), PT (+++), OL (+), periplaque (+), DOL (+++)	OL (+++), EN‐L2‐3 (+), EN‐L5‐6
IL1RAPL2	Exc RORB_C (+), Exc_RORB_A (++), Exc_RORB_B (+++), Exc_THEMIS_RORB (+++), Inh_VIP_B (+) Inh_RELN_A (+++), Inh_RELN_B (+++)	PT (++)	EN‐L4
IL4R	Astro_Mixed (, +++), Micro_A (HM, +), Micro_C/E (MIMS, +), Micro_Prolif (+) EC_Capillary (+), EC_Venous (+++) Endo_PVM (+)	AIMS (+), PI (+++), chronic active edge (+), perivasc Mac (++)	Endo cells (++)
IL6	Astro A (+++), Astro F (RA, +++), Micro A (HM, +), Micro C (MIMS, +)		
IL6R	Astro_A (AGC, +), Astro_F (RA, +++), Astro_Mixed (+) Micro_A/B (HM, +), Micro_D/E (MIMS, +) EC_Venous (+++), Perivascular_Fibroblast (+) Endo_PVM (+)	Perivasc Mac (+)	
TNF	Micro_A/B/C/D (+), Micro_E (++) Micro_Prolif (+++)		
TNFRSF1A	Astro_C (+), Astro_D/E/F (RA, +), Astro_Mixed (+) Micro_A (HM, +), Micro_C (MIMS, +) EC_Venous (+), EC_Arterial (+), EC_Stressed (+), Pericyte (+), Endo_PVM (+), Arterial_Smooth_Muscle_Cell (+)		
TNFRSF1B	Astro_Mixed (+++), Micro_C (MIMS, +) PVM (+), Endocyte_Venous (+), Pericyte (+), Endo_PVM (++), PT (+)	Demyelinating OL (++), chronic active edge (++)	
TNFRSF21	OPC_1 (++), COP (+++), Oligo_D (+++), Exc_THEMIS_A (+), Exc_TLE4_B (+), Pericyte (+++)	PT (+), perivasc Mac (+)	
TNFSF13B (BAFF/BLyS)	Micro_C/D/E (MIMS, +)	Periplaque (+)	
Complement factors			
C1q	C1QA: Micro_A (HM, +), Micro_C/E (MIMS, +), Micro_Prolif (+), PVM (+), Endo_PVM (+++) C1QB: Micro_A (HM, +), Micro_C/E (MIMS, +) Micro_E (MIMS, +), Micro_Prolif (+), Micro_Astro (HM, +), Endo_PVM (+++) C1QC: Micro_A (HM, +), Micro_C/E (MIMS, +), Micro_Prolif (+), Endo_PVM (+++)	C1QA: OL (++) C1QB: AIMS (+++), MIMS (++), OL (++), chronic active edge (+), HM (+) C1QC: AIMS (++), MIMS (+), OL (+)	C1QA: Microglia (+++) C1QB: Microglia (+++) C1QC: Microglia (++)
C3	Astro_D (RA, +++), Astro_E (RA, +), Astro_Mixed (++), Micro_A/B (HM, +), Micro_E (MIMS, +), Micro_Prolif (+)	AIMS (+++), MIMS (++), OPC (+), control white matter (+)	Phagocytes (++), Microglia (+++)
TLR			
TLR1	Micro_A/B (HM, +), Micro_B (+), Micro_C/E (MIMS, +)		
TLR2	Micro_A (HM, +), Micro_C/E (MIMS, +) PVM (+), Endo_PVM (+++)	AIMS (+++), MIMS (+++), astrocytes/glial cells (++), OL (++), chronic active edge(+)	
TLR5	Micro_A/B (HM, +)		
TLR7	Micro_A (HM, +), Micro_E (MIMS, +)		
TLR10	Micro_A (HM, ++)		

*Note*: Absinta et al. (NIHMS1762170‐supplement‐Supplementary_Tables), Macnair et al. (data_S4B_marker_genes_fine_cell_type), and Schirmer et al. (41586_2019_1404_MOESM1_ESM). “+++” (Strong Upregulation) → logFC ≥ 1.5. “++” (Moderate Upregulation) → 1.0 ≤ logFC < 1.5. “+” (Mild Upregulation) → logFC < 1.0.

Abbreviations: AGC, astrocytes/glial cells; AIMS, inflamed astrocytes; Astrocytes, astrocyte cells (general category); BP, B cells/plasmablasts; COP, committed oligodendrocyte precursor cell; DOL, demyelinating oligodendrocytes; EN‐MIX, excitatory neurons mixed; Exc, excitatory; FLC, fibroblast‐like cells; HM, homeostatic microglia; IN‐PVALB, parvalbumin‐positive inhibitory interneurons; IN‐SST, somatostatin‐positive inhibitory interneurons; IN‐SV2C, synaptic vesicle glycoprotein 2C‐positive inhibitory interneurons; IN‐VIP, vasoactive intestinal peptide‐positive inhibitory interneurons; MDC, monocytes/dendritic cells; MIMS, microglia inflamed in MS; MOL, mature oligodendrocytes; OL, oligodendrocytes; OPC, oligodendrocyte precursor cells; Phagocytes, phagocytic immune cells (including macrophages, dendritic cells); PI, peripheral immune cells; PT, T lymphocytes; PVM, perivascular macrophages; RA, reactive astrocytes; Stromal cells, connective tissue‐supporting cells; VC, vascular cells.

### 5.9 Glial Dysfunction and Aging

Aging also emerges as a central factor in MS pathophysiology. The most significant risk factor for disability in MS is age: younger patients are more likely to experience symptom remission, whereas strongly disabling deficits typically appear after the 4th decade of life (Confavreux and Vukusic 2006). This aligns with the broader phenomenon of aging glial cells contributing to neurodegenerative disorders (Palmer and Ousman 2018; Streit et al. 2020). Aging appears to impair the CNS's capability to recycle proteins and other molecules, leading to the accumulation of byproducts such as iron, which is particularly prominent in progressive MS lesions (Rudko et al. 2014; Dal‐Bianco et al. 2021). Histopathological studies have shown that iron accumulation in lesions is associated with abnormalities in astrocytes and microglia, pointing to these cell subsets as critical players in the chronicity of the disease (Popescu et al. 2017). An open question is whether iron deposition is merely an epiphenomenon of chronic inflammation or an active driver of MS pathophysiology. However, the correlation between aging, disability, and iron accumulation suggests at least a bystander role.

## 6 Molecular Overlap Between the CNS and Immune System: Dual‐Use Pathways in MS Pathophysiology

Transcriptomic analyzes in MS (Table 1) showed that microglia and reactive astrocytes strongly upregulate cytokines such as IL‐1β and IL‐6, alongside TLR2, which suggests innate immune activation in chronic lesions. Complement activation is also a prominent feature, with C1QA, C1QB, and C1QC highly expressed in microglia and inflamed astrocytes, while C3 is upregulated in astrocytes and OPCs, reinforcing their involvement in neuroinflammatory signaling. Moreover, MHC class I genes (HLA‐A, HLA‐B, HLA‐C, HLA‐E) are upregulated in OPCs, astrocytes, and vascular cells, whereas MHC class II genes (HLA‐DPA1, HLA‐DPB1, HLA‐DRB1) are primarily found in microglia, perivascular macrophages, and endothelial cells. Chemokines such as CXCL10 and CX3CL1 are differentially expressed, with CXCL10 highly enriched in microglia and CX3CL1 in astrocytes, supporting the recruitment and activation of immune cells in lesion environments. Furthermore, growth factors and their receptors, including VEGFA (upregulated in OPCs and reactive astrocytes) and TGFBR1 (highly expressed in OPCs and inflamed astrocytes) highlight the potential for both repair and pathogenic remodeling of the CNS. Notably, studies of remyelinating lesions have identified molecules such as CXCL12, EGF, and IL‐10 as potential drivers of efficient remyelination, with upstream regulators including TGFB1, TGFB2, EGF, and IGF implicated in orchestrating these regenerative processes (Chen et al. 2024).

Collectively, these findings underscore the central role of multiple glial populations in MS pathophysiology, revealing a complex interplay between immune activation, complement signaling, and glial remodeling. Remarkably, the data supporting these conclusions are derived from post‐mortem tissue of individuals with long‐standing MS, and they illustrate that in the later stages of the disease, glial‐driven inflammatory responses clearly predominate over peripheral immune cell involvement. This is further supported by the notably low abundance of peripheral immune cells detected in isolated cells from these tissues. These late‐stage snapshots of MS emphasize that, over time, inflammatory activity within the CNS becomes self‐sustaining, reducing or even eliminating the need for continued peripheral immune input. However, the molecular and cellular mechanisms that initiate and perpetuate this chronic state of glial inflammation remain largely unresolved, representing a critical gap in our understanding of progressive MS.

Understanding why inflammation in MS fails to resolve and instead becomes chronic requires stepping back to consider broader systemic interactions. The harmonious interaction of specialized biological systems is a challenging task and represents a major point of vulnerability to emergent disease processes. The immune system and the CNS are two of the most intricate systems in mammals. The immune system operates dynamically, with blood‐borne forces surveying the entire body, from lymphoid organs to peripheral tissues, to identify and neutralize threats (Dahlgren and Molofsky 2019). These patrolling units are equipped with potent effector mechanisms designed to ensure the body's integrity. In contrast, the CNS appears macroscopically stable but exhibits intricate microstructural dynamics (Dekkers et al. 2013; Allen and Lyons 2018). It must balance providing a dependable framework for motor and bodily functions while maintaining the plasticity needed for cognitive processes over a lifetime.

The unique susceptibility of humans to MS may stem from evolutionary adaptations of the human CNS, particularly in myelination and synaptic plasticity, which enabled higher cognitive functions and lifelong adaptation of the neuronal network (Miller et al. 2012; Sherwood and Gómez‐Robles 2017; Goldman 2020). Evidence suggests that molecules and pathways have been employed parallelly but independently in the immune system and the CNS. Growth factors, cytokines, chemokines, MHC, toll‐like receptors, and the complement system have crucial roles not only in the immune system for defense purposes but also in the nervous system for non‐immune functions, e.g., synapse pruning, myelination, and demyelination. This repurposing of similar molecules is in the following referred to as “dual‐use tools”. While these tools generally function independently, disruptions can lead to misapplications, particularly in individuals with underlying susceptibilities.

### 6.1 MHC Molecules

MHC‐I family members are critical for CNS processes such as synapse pruning, myelination, and demyelination. Studies in knockout mice have shown that neuronal expression of MHC‐I is essential for synapse elimination during early neuronal circuit development (Lee et al. 2014). In adult brains, MHC‐I expression is inducible, influenced by neural activity and cytokines like interferon‐gamma (IFN‐γ), which facilitates immune surveillance and potential antigen presentation that may lead to cytotoxicity by CD8+ T cells (Corriveau et al. 1998; Medana et al. 2001; Cebrián et al. 2014). Transcriptomic data reveal that both class I (HLA‐A, ‐B, ‐C, ‐E) and class II (HLA‐DPA1, ‐DPB1, ‐DQB1, ‐DRA, ‐DRB1) molecules are expressed not only by infiltrating immune cells but also by glial, endothelial, and oligodendroglial cells. Notably, astrocytes and microglia show strong expression of HLA class II genes, suggesting potential for local antigen presentation and T cell interaction in MS lesions. HLA‐E, a non‐classical class I molecule, is prominently expressed in reactive astrocytes and vascular cells, implicating it in the regulation of immune tolerance and NK cell activity. The presence of HLA class I molecules on OPCs and endothelial cells points to their involvement in both immune signaling and possibly non‐immune processes such as cell communication or differentiation.

### 6.2 Chemokines

Chemokines, another group of dual‐use molecules, are critical for immune cell migration and CNS development. CXCR4 and its chemokine stromal derived factor (SDF‐1) guide neuronal and glial cell positioning (Vilz et al. 2005), while CXCR2 regulates OPC positioning and myelination in the developing brain and controls the positioning and arrest of these cells and by that induces proper myelination (Padovani‐Claudio et al. 2006). Interestingly, blocking CXCR2 enhances OPC remyelination in demyelinating models (Liu et al. 2010). CX3CR1 and its ligand CX3CL1 (fractalkine) mediate microglia expansion and neuronal synapse pruning in the developing brain (Zhan et al. 2014). In the peripheral immune system, fractalkine and its receptor are relevant for homeostatic resident phagocytes in non‐lymphoid tissues, such as macrophage populations in non‐lymphoid tissues (Geissmann et al. 2003).

### 6.3 Growth Factors and Cytokines

Growth factors, such as ciliary neurotrophic factor (CNTF), belong to the IL‐6 family and demonstrate dual roles. In the CNS, CNTF supports motor neuron survival, while in the immune system, it promotes hematopoiesis and B cell generation (Sendtner et al. 1994; Askmyr et al. 2015). In MS, CNTF expression is elevated in glial cells and cortical neurons compared to healthy controls (Dutta et al. 2007). Neurotrophins, including nerve growth factor (NGF) and brain‐derived neurotrophic factor (BDNF), are also implicated in inflammation, though their precise roles remain unclear (Vega et al. 2003). In particular, NGF has been reported to be increased in the serum of patients with rheumatoid arthritis (Rihl et al. 2005) but also allergic disease (Bonini et al. 1996).

Based on the data provided in Table 1, a variety of cell types express specific growth factors and their corresponding receptors, which play crucial roles in cellular signaling and tissue regulation, especially in neurological and immune environments. For instance, Brain‐Derived Neurotrophic Factor (BDNF) is strongly expressed by Exc_THEMIS_B neurons, highlighting its role in promoting neuronal survival and plasticity.

Fibroblastic growth factors (FGFs) and their receptors have been implicated in CNS development (Reuss and von Bohlen und Halbach 2003) as well as diverse neurological diseases, e.g., episodic ataxia (FGF14‐related SCA‐27B) and FGF12, which has been reported in Alzheimer's disease (AD). In the transcriptomic analyzes of MS brain tissue (see Table 1), some of these factors (FGF12, FGF14) are regulated in the AIMS astrocyte population as well as in diverse other immune and CNS cell populations. The receptor FGFR1 was predominantly upregulated on OPCs, astrocytes, excitatory and inhibitory neurons, and various immune cells. Oligodendrocyte‐specific FGFR1‐KO in EAE was reported to result in reduced inflammation, demyelination, and axonal damage (Rajendran et al. 2021) and a study in humans identified FGF5 and FGF10 as potential pro‐remyelinating molecules (Chen et al. 2024). Furthermore, a pro‐inflammatory role via FGFR1 on microglia and IL‐6 induction has been suggested in the context of borreliosis in an in vitro macaque‐derived microglia culture model (Parthasarathy et al. 2023), the latter showing the close interconnection of growth factors with cytokines.

Transforming Growth Factor‐beta 1 (TGF‐β1) exemplifies growth‐factor‐/cytokine‐mediated communication between astrocytes, microglia, and neurons. TGF‐β1 modulates astrocyte and microglial function, promoting neuroprotection and regulating inflammatory responses (Unsicker et al. 1991; Buckwalter and Wyss‐Coray 2004; Koeglsperger et al. 2013) with more refined expression patterns observed in primates (Li et al. 2023). The absence of CNS TGF‐β1 expression in mice results in impaired glutamate removal due to reduced expression of astrocytic glutamate transporters (Koeglsperger et al. 2013). In this mouse model, microglia numbers were initially reduced and continued to decline throughout adulthood, leading to motor abnormalities and early death associated with severe microglial dysfunction (Butovsky et al. 2014). Additionally, TGF‐β1 has been implicated in promoting microglial neuroprotective functions by reducing glial activation in the context of amyloid pathology (Wyss‐Coray et al. 2001; Chen et al. 2015) and is involved in key astrocyte reactivity programs (Barkhuizen et al. 2022). In CNS cell culture models of MS, astrocyte‐derived TGF‐β1 was observed in benign forms of the disease but was notably absent in progressive MS, underscoring its potential neuroprotective function (Kerkering et al. 2023). In MS tissue transcriptomics, TGFBR1, TGFBR2, and TGFBR3 show a broad expression across immune and glial populations. TGFBR1 is strongly upregulated in committed oligodendrocyte precursors (COP), microglia, and astrocytes, reflecting its regulatory role in inflammation and repair. TGFBR2 and TGFBR3 also show widespread expression in astrocytes, microglia, vascular, and stromal cells. Of the TGFB ligands, TGFB1 and TGFB2 are found in vascular cells and astrocytes, reinforcing their involvement in maintaining the blood–brain barrier and immune modulation (Table 1).

Vascular Endothelial Growth Factors (VEGFA and VEGFC) are also widely expressed. VEGFA is notably high in OPCs, astrocytes, and demyelinating oligodendrocytes (DOL), reflecting its role in angiogenesis and neuroinflammation. VEGFC, similarly, is expressed in vascular and smooth muscle cells.

Insulin‐like Growth Factor Binding Proteins (IGFBPs) such as IGFBP2, IGFBP3, IGFBP4, IGFBP5, and IGFBP7 are expressed in perivascular fibroblasts, arterial muscle cells, stromal, and immune cells. They modulate IGF availability and influence processes like growth, survival, and inflammation. Finally, EGF and its receptor EGFR are prominently expressed in reactive astrocytes, OPCs, and multiple inhibitory interneurons, indicating their function in glial proliferation and neuron–glia signaling. Overall, these growth factors and their expression profiles underscore a complex interplay of neuronal, glial, vascular, and immune cells in both homeostatic and pathological brain environments.

The cytokine IL‐1β is constitutively expressed at very low levels in the human brain and frequently upregulated in the context of neurodegeneration and inflammation (Allan et al. 2005). IL‐1β plays a key role in initiating the production of other inflammatory cytokines and chemokines, with the term ‘neuroinflammation’ often used to describe the presence of IL‐1β in the context of microglial activation (Liu and Quan 2018). Increased protein levels of IL‐1β have been detected in CNS lesions of pwMS (Lin and Edelson 2017), with most pronounced regulation in reactive astrocytes (Table 1). Besides their context in inflammation and neurodegeneration, autocrine IL‐1R signaling on microglia seems also crucial for self‐renewal and proliferation of microglia (Bruttger et al. 2015). Other cytokine receptors identified in the transcriptomic approach the anti‐inflammatory IL‐4 and IL‐10 receptors (expressed on glial cells as well as immune cells). The potential role of IL‐4R for neuroregeneration has been well documented (Walsh et al. 2015). Furthermore, glial cells (mainly the microglia) have been identified to produce the T cell survival cytokine IL‐15 and the pro‐inflammatory IL‐18. Also, cytokine receptors IL1R1, IL18R1, IL17RA, IL17RB and the TNF receptors are regulated in MS lesions (Table 1). Notably, we previously reported that human iPSC‐derived neurons express IL17RA and TNF receptors, which sensitized them to cytokine‐induced axonal damage (Meyer‐Arndt et al. 2023). In mice, IL17RA was predominantly expressed in hippocampal neurons; although its physiological role remains unclear, IL‐17A overexpression resulted in impaired synaptic plasticity and learning deficits (Di Filippo et al. 2021).

These findings support the notion that cytokine receptors serve as true dual‐purpose mediators, with distinct yet potentially intersecting roles in both CNS development and neuroinflammatory processes. In this context, a deeper understanding of the functions of these cytokines and their receptors in human brain development and adult brain homeostasis is essential for unraveling the mechanisms underlying their dysregulation in multiple sclerosis.

### 6.4 Toll‐Like Receptors (TLR)

TLRs are innate immune receptors mediating responses to pathogen‐associated molecular patterns (PAMPs) and danger‐associated molecular patterns (DAMPs). This is another example of a genuine dual‐use tool as this immune‐attributed receptor type has been shown to be expressed in distinct embryonal, fetal, and postnatal phases of brain development (Kaul et al. 2012). The function to remove potentially dangerous molecules is used for developmental processes where large amounts of apoptotic cells have to be removed (e.g., brain development) as well as in sensing CNS‐entering pathogens extra‐ and intracellularly. The major cellular player using TLRs is therefore the microglia (Abarca‐Merlin et al. 2024). However, all other CNS cells, including astrocytes and neurons, have been shown to employ these receptors for specific purposes. In experimental models, the involvement of TLR2 and TLR7 has been shown to mediate neurodegenerative responses (Lehnardt 2010). In MS lesions (Table 1), the predominant cell set expressing these receptors is microglia, with TLR2 being the most prominently expressed TLR in the chronic mixed active/inactive lesion edge (in AIMS, MIMS and oligodendrocytes). Furthermore, TLR2 has been investigated as being involved in failed remyelination in MS (Sloane et al. 2010)

### 6.5 Complement System

Complement proteins, such as C3, are critical for synapse pruning in the CNS and immune defense in the periphery (Stephan et al. 2012; Coulthard et al. 2018). Microglia use complement proteins like C3b during myelin removal in mixed active/inactive MS lesions (Prineas et al. 2001; Ramaglia et al. 2012). Complement‐mediated synapse loss is a hallmark of MS progression, though the mechanisms behind persistent complement deposition remain unclear (Werneburg et al. 2020). Evidence of microglia‐astrocyte interactions—involving C1q—further underscores the complexity of glial activation in MS (Liddelow et al. 2017) and highlights the complex interplay between these two glial cell types in inflammatory conditions. The transcriptional modulation of complement proteins has emerged as a defining feature of dysregulated microglia and astrocytes in MS lesions, further emphasizing their role in the inflammatory and degenerative processes associated with the disease (Absinta et al. 2021) and was confirmed in the large transcriptomic analysis by Macnair and colleagues (Table 1).

### 6.6 Implications for Chronic CNS Inflammation in MS

The dual‐use nature of molecular tools, essential for both CNS maintenance and immune defense, creates vulnerabilities when CNS‐peripheral immune communication is disrupted. This could be particularly relevant in humans, where these processes are not restricted to differential periods but occur at the same time. Notably, recent findings highlight extensive C1q‐mediated microglial engulfment of synapses in hippocampal regions of individuals with MS exhibiting cognitive impairment (Barros et al. 2024). Misinterpretation of homeostatic cues by glial cells and peripheral immune cells may perpetuate inflammation, leading to chronic neurodegeneration. Identifying CNS‐specific aberrations and understanding how they interact with peripheral immune responses are critical for elucidating the mechanisms driving progressive MS. Given their persistent expression in progressive MS lesions and their diverse regulatory roles, these molecules (Table 1) represent promising targets for future CNS‐specific interventions. This research provides a foundation for targeting glial and immune interactions to develop novel therapeutic approaches for MS.

## 7 Future Directions: Open Questions and Research Opportunities

1. Understanding Persisting Lesions in MS
  - Are there epigenetic alterations in glial cells, particularly oligodendrocytes, microglia, and astrocytes, that prevent the successful regeneration of myelin sheaths in demyelinated plaques?
  - Could these epigenetic changes be induced or maintained by chronic inflammatory conditions within the CNS, and if so, what are the signaling pathways or molecular mediators involved?
  - Are tissue resident lymphocytes (even as very rare subset) relevant?
2. Dual‐Use Tools in MS pathology
  - How do molecules such as MHC, complement factors (e.g., C1q, C3), and cytokines influence glial cell behavior in the CNS under both homeostatic and inflammatory conditions?
  - Are these tools misapplied in MS, leading to maladaptive interactions between glial cells and peripheral immune cells?
  - What are the key mechanisms by which these molecules mediate synapse loss, myelin stripping, and glial activation in progressive MS?
3. Chronic Inflammation and Neurodegeneration
  - What are the specific roles of microglia and astrocytes in maintaining chronic inflammatory states in MS lesions?
  - Do microglia and astrocytes actively drive neurodegeneration in progressive MS, or are they reactive participants in response to signals from other cell types?
  - How do glial‐glial and glial‐neuronal interactions evolve in MS lesions, and can disrupting these maladaptive networks halt disease progression?
4. Environmental Factors and MS Pathology
  - How do environmental factors such as vitamin D deficiency, EBV infection, smoking, air pollution, and dietary influences impact the functional state of glial cells, particularly microglia, astrocytes, and oligodendrocyte lineage cells?
  - Do these environmental factors induce lasting epigenetic changes within glial populations, altering their inflammatory or regenerative capacities in MS lesions?
  - Can identifying and targeting environmentally induced molecular pathways in CNS‐resident cells offer novel preventive or therapeutic approaches for MS?
5. Impact of Aging
  - How does aging affect the regenerative potential of glial cells, particularly OPCs and astrocytes?
  - Is the age‐related accumulation of iron in MS lesions a driver of glial dysfunction, or is it merely a marker of disease chronicity?
  - Are there distinct molecular signatures of glial aging that can be targeted to improve repair processes in MS?
6. Targeting Glial Pathology in MS Therapy
  - Can molecular pathways specific to glial cells be modulated to enhance remyelination or suppress maladaptive inflammatory states?
  - Are there therapeutic opportunities in manipulating the complement system or other signaling pathways shared between the CNS and immune system to reduce synapse loss and neurodegeneration?
  - How can advanced single‐cell sequencing, epigenetic characterization, and imaging techniques be used to identify novel therapeutic targets within glial populations?

Future research must leverage insights gained from detailed characterization of late‐stage lesion pathology to uncover the precise mechanisms underpinning chronic glial inflammation in MS. Elucidating the initiating factors and ongoing mechanisms that perpetuate this persistent inflammatory state is critical, as they represent not only the core of MS pathology but also key opportunities for therapeutic intervention. Particular emphasis should be placed on deciphering the molecular and epigenetic alterations in glial cells that obstruct effective remyelination and drive neurodegeneration. While sequencing technologies yield numerous potential therapeutic targets, prioritizing dual‐use molecules might be especially advantageous. These molecules, uniquely functioning within both immune and nervous systems, may have evolved recently in humans, potentially explaining the human‐specific high prevalence of MS. Utilizing advanced humanized experimental models will be crucial to accurately simulate and dissect the complex cellular interactions and inflammatory processes. Such methodologies promise to significantly enhance our ability to identify novel therapeutic avenues, ultimately enabling restoration of glial homeostasis, modifying disease trajectories, and improving clinical outcomes for patients with progressive MS.

## Author Contributions

V.S. conceptualized and wrote the manuscript.

## Conflicts of Interest

The author declares no conflicts of interest.

## Data Availability

Data sharing not applicable to this article as no datasets were generated or analysed during the current study.
